# Coupling between skeletal muscle fiber size and capillarization is maintained during healthy aging

**DOI:** 10.1002/jcsm.12194

**Published:** 2017-04-05

**Authors:** Yoann Barnouin, Jamie S. McPhee, Gillian Butler‐Browne, Alessandra Bosutti, Giuseppe De Vito, David A. Jones, Marco Narici, Anthony Behin, Jean‐Yves Hogrel, Hans Degens

**Affiliations:** ^1^ School of Healthcare Science Manchester Metropolitan University Chester Street Manchester M1 5GD UK; ^2^ Institut de Myologie, UPMC UM 76, INSERM U 974, CNRS UMR 7215 Pitle‐Salpetriere Hospital 47‐83 Boulevard de l'Hopital 75013 Paris France; ^3^ Istituto di Anatomia Patologica, Dipartimento di Scienze Mediche, Chirurgiche e della Salute University of Trieste, Cattinara Hospital Strada di Fiume 447 34149 Trieste Italy; ^4^ Physiotherapy & Sports Science, Health Sciences Centre School of Public Health Belfield Dublin 4 D04 V1W8 Ireland; ^5^ Division of Medical Sciences & Graduate Entry Medicine, School of Medicine, Faculty of Medicine & Health Sciences, MRC‐ARUK Centre of Excellence for Musculoskeletal Ageing Research, Derby Royal Hospital University of Nottingham Uttoxeter Road Derby DE22 3DT UK; ^6^ AP‐HP—Centre de Référence de Pathologies Neuromusculaire Paris Est—Institut de Myologie Paris France; ^7^ Lithuanian Sports University 6 Sporto St LT‐44221 Kaunas Lithuania

**Keywords:** Ageing, Muscle fibre, Capillary, Succinate dehydrogenase, Oxidative capacity

## Abstract

**Background:**

As muscle capillarization is related to the oxidative capacity of the muscle and the size of muscle fibres, capillary rarefaction may contribute to sarcopenia and functional impairment in older adults. Therefore, it is important to assess how ageing affects muscle capillarization and the interrelationship between fibre capillary supply with the oxidative capacity and size of the fibres.

**Methods:**

Muscle biopsies from healthy recreationally active young (22 years; 14 men and 5 women) and older (74 years; 22 men and 6 women) people were assessed for muscle capillarization and the distribution of capillaries with the method of capillary domains. Oxidative capacity of muscle fibres was assessed with quantitative histochemistry for succinate dehydrogenase (SDH) activity.

**Results:**

There was no significant age‐related reduction in muscle fibre oxidative capacity. Despite 18% type II fibre atrophy (*P* = 0.019) and 23% fewer capillaries per fibre (*P* < 0.002) in the old people, there was no significant difference in capillary distribution between young and old people, irrespective of sex. The capillary supply to a fibre was primarily determined by fibre size and only to a small extent by oxidative capacity, irrespective of age and sex. Based on SDH, the maximal oxygen consumption supported by a capillary did not differ significantly between young and old people.

**Conclusions:**

The similar quantitative and qualitative distribution of capillaries within muscle from healthy recreationally active older people and young adults indicates that the age‐related capillary rarefaction, which does occur, nevertheless maintains the coupling between skeletal muscle fibre size and capillarization during healthy ageing.

## Introduction

The microcirculation plays a crucial role in the delivery of oxygen, nutrients, and hormones to, and removal of heat, metabolites, and waste products from, muscle fibres. In line with the idea that the main role of capillaries is oxygen delivery, oxidative muscles with a large maximal oxygen demand have a higher capillary density than glycolytic muscles.^1^, ^2^, ^3^ Even at the level of the single fibres, a positive relationship between the mitochondrial volume density and number of capillaries supplying a fibre has been reported.^4^ Other studies have shown that the number of capillaries per fibre is also positively related to fibre size.^5^, ^6^, ^7^ The coupling between fibre size and capillaries per fibre is further emphasized by the similar time course of hypertrophy and angiogenesis during the development of hypertrophy.^8^ Given these observations, one might expect that the age‐related decreases in fibre size and oxidative capacity are associated with capillary rarefaction.

So far, studies have shown that the capillary density is largely maintained during ageing, indicating that capillary rarefaction, as reflected by a reduction in the capillary‐to‐fibre ratio, is proportional to the decrease in fibre size.^9^ However, the slope of the relationship between the number of capillaries supplying a fibre and the size of the fibre was slightly reduced in old rats,^5^ possibly because of the lower oxidative capacity of the old rat muscle.^10^ Even so, some studies indicate a superfluous capillary supply in old rodent muscles, where there were no differences in capillary density and capillary‐to‐fibre ratio, despite a reduction in the oxidative capacity,^11^ or even a higher capillary density, with similar oxidative capacity, than in young rats.^12^ There are indications that these relationships may also change in human muscle as the relationship between maximum oxygen uptake and oxygen kinetics with capillarization in young people has disappeared in old men.^13^, ^14^

Not only the number of capillaries per fibre and fibre area are important for tissue oxygenation but also the way capillaries are distributed, where a heterogeneous distribution of capillaries has a negative impact on tissue oxygenation.^15^, ^16^, ^17^, ^18^ Indeed, model calculations indicate that random blockage of capillaries, resulting in an increased heterogeneity of the distribution of perfused capillaries during sepsis, contributes to the ensuing muscle pathology.^19^ In rats, there is some indication that the heterogeneity of capillary spacing increases with age, which appeared to be related to the increased heterogeneity in fibre size.^20^ While it is likely that the increased heterogeneity in fibre sizes is the consequence of a denervation–reinnervation process during ageing,^21^ the associated increase in the heterogeneity of capillary spacing may accelerate the development of age‐related muscle wasting. If such a situation also occurs in human muscle, it may adversely affect muscle oxygenation, as well as the removal of metabolites and heat from active skeletal muscles, and thereby contribute to the age‐related reduction in physical performance.

The aim of the present study was to compare overall capillarization and capillary supply of individual fibres in muscles from young and old men and women. To investigate this, we calculated capillary domains as the areas surrounding a capillary delineated by equidistant boundaries from adjacent capillaries,^22^ which is an index of the oxygen supply area of a capillary, including in muscles with a heterogeneous fibre type composition.^23^ Quantitative succinate dehydrogenase (SDH) histochemistry was used to estimate the maximal oxygen consumption of a fibre,^24^ to determine (i) the relationship between the oxidative capacity and the capillary supply to a fibre and (ii) whether this relationship is different in older human muscle compared with young muscle. We hypothesized that compared with young muscle, older muscle would have (i) lower capillary density, indicative of capillary rarefaction exceeding fibre atrophy; (ii) greater heterogeneity of capillary spacing; and (iii) a capillary supply to a fibre in excess of its oxidative capacity due to a proportionally larger loss in fibre oxidative capacity.

## Methods

### Subject characteristics and biopsy sampling

Forty‐seven healthy men and women were recruited to study the effects of ageing on muscle morphology (*Table* 1). The local ethics committees of Manchester Metropolitan University (United Kingdom) and of Ile‐de‐France VI in Paris (France) approved the study, and all participants provided written informed consent. All experiments have been performed in accordance with the ethical standards laid down in the 1964 Declaration of Helsinki and its later amendments. The older participants in the study were healthier than typical for their age,^25^ and subjects suffering from known cardiovascular, neuromuscular, or respiratory diseases were excluded. Thirty‐five participants completed a questionnaire to generate a habitual physical activity score,^26^ where scores <6 represent a sedentary lifestyle and >9 indicate a high level of physical activity. Vastus lateralis muscle biopsies were taken midway between the patella and greater trochanter under aseptic conditions with either a conchotome or Bergström needle after local anaesthesia with 2% lidocaine. The muscle sample was placed on cork with Optimum Cutting Temperature compound (Scigen® Gardena) and rapidly frozen in isopentane cooled in liquid nitrogen, or with vigorous shaking in liquid nitrogen and stored at −80°C until analysis.

**Table 1:** Participant characteristics

	Young		Old		Effects (*P*‐values)		Interactions (*P*‐values)
	Men, *N* = 14	Women, *N* = 5	Men, *N* = 22	Women, *N* = 6			
					Age	Sex	AS
Age (years)	22.1 ± 2.9	21.0 ± 2.4	73.5 ± 3.9	74.5 ± 3.7	<0.0005	0.990	0.386
Height (m)	1.78 ± 0.06	1.64 ± 0.03	1.72 ± 0.07	1.60 ± 0.05	0.012	<0.0005	0.716
Body mass (kg)	70.7 ± 10.8	61.1 ± 9.0	81.6 ± 14.4	60.4 ± 6.0	0.241	0.001	0.176
BMI (kg · m^‐2^)	22.3 ± 2.6	22.6 ± 2.9	27.6 ± 3.4	23.7 ± 1.9	0.004	0.101	0.051
PAS	9.8 ± 1.0	9.7 ± 1.5	8.3 ± 1.6	8.7 ± 1.5	0.054	0.875	0.676

AS, age × sex interaction; BMI, body mass index; PAS, physical activity score (available for 35 participants; 6 young men, 3 young women, 20 old men, and 6 old women).

Values are presented as mean ± standard deviation.

### Histochemistry

**Figure 1:**
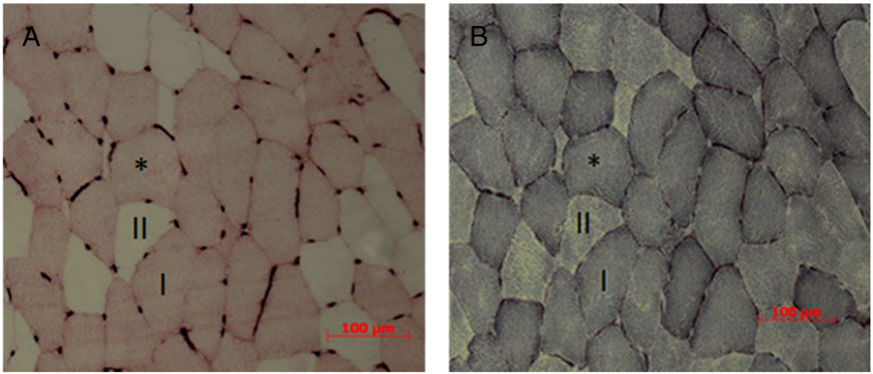
Typical example of serial vastus lateralis muscle sections from an old man stained for (A) myosin heavy chain (MHC) type I (dark stained) and capillaries (dark dots), (B) succinate dehydrogenase (SDH) activity. Note that type I fibres (dark stained) had, as expected, a higher SDH activity compared with type II fibres (light stained). Asterisk (*) identifies same fibre in the two panels.

Serial 10 μm cross‐sections of the vastus lateralis muscle biopsies were cut in a cryostat and capillaries and type I fibres co‐stained (*Figure* 1A) as described previously.^2^, ^27^ Briefly, sections were dried and fixed in ice‐cold acetone for 5 min and after washing in 2‐[4‐(2‐hydroxyethyl)piperazin‐1‐yl]ethanesulfonic acid (HEPES) buffer, the sections were blocked in 0.1% bovine serum albumin in HEPES for 60 min. After 15 min peroxide incubation, the sections were incubated with Ulex europaeus Agglutinin I lectin (50 μg mL^−1^) in 1% bovine serum albumin‐HEPES combined with anti‐Myosin Heavy Chain type I [0.41 μg mL^−1^, Developmental Studies Hybridoma Bank (DSHB, USA)] for 1 h to detect capillary locations and type I fibres, respectively. Sections were then incubated with a secondary ‘Vectastain anti‐mouse IgG antibody’ (Vector Laboratories, Peterborough, UK) and stained using the ‘Vectastain ABC’ kit (Vector Laboratories). Finally, type I fibres were visualized by incubation in the peroxidase substrate ‘Vector VIP’ kit (Vector Laboratories). The sections were mounted in glycerol‐gelatin for further analysis.^2^, ^27^

A serial section was stained for SDH (*Figure* 1B), as a marker of fibre oxidative capacity.^2^, ^7^, ^27^ Briefly, the section was dried for 15 min and then incubated at 37°C in the dark for 20 min in a medium consisting of 0.37 M sodium phosphate buffer (pH 7.6), 74 mM sodium succinate, and 0.4 mM tetranitroblue tetrazolium. The reaction was stopped with a 30 s incubation in 0.01 M Hydrogen chloride, washed with distilled water, and mounted in glycerol‐gelatin.

### Morphometry

**Figure 2:**
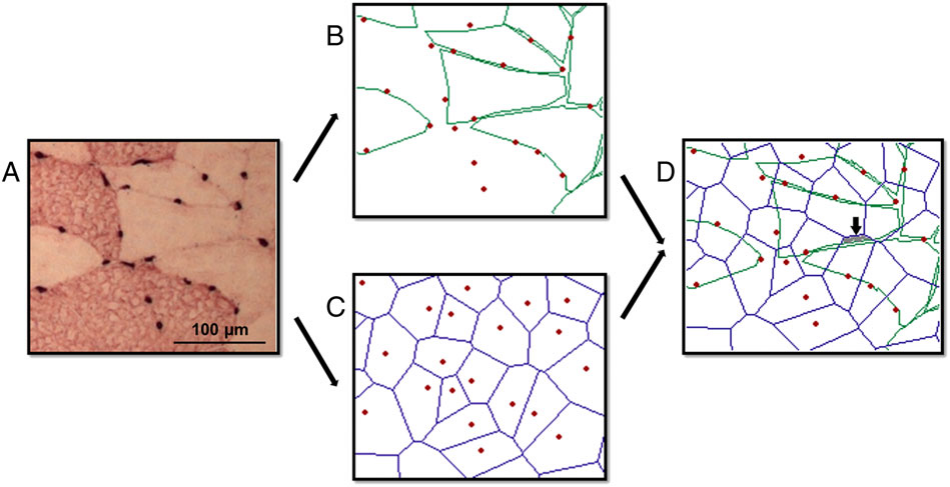
Fibre outlines and capillary domain areas. In (A), an example of a small part of a muscle section from a young man stained for myosin heavy chain type I (type I fibres appear dark and type II fibres light) and capillaries (black dots around the fibres). In (B), the type II fibre outlines are shown with the capillaries as red dots. In (C), the capillary domains are illustrated; the contours indicate the borders of the capillary domains, and the red dots correspond to the capillaries. In (D), the overlap of capillary domains and type II fibres is illustrated. It is important to note that a fibre may receive oxygen also from capillaries not in direct contact with the fibre; this situation occurs when a fibre overlaps a domain from a non‐adjacent capillary (in grey, indicated by the arrow).

Fibre outlines on printed images (*Figure* 2A) were traced on a digitizing tablet (model MMII 1201, Summagraphics, Austin, TX, USA), and the co‐ordinates of the outline stored for further analysis with AnaTis (BaLoH software, NL) (*Figure* 2B). The variation in fibre cross‐sectional area (FCSA) was given as the standard deviation of the FCSA (SD FCSA). Roundness was calculated as follows^27^: perimeter^2^/(4π·FCSA); increasing values indicate increasing deviation from circularity (irregularities). Fibre type proportions were expressed as (i) the fibre number percentage, and (ii) the fibre area percentage (FAP) expressed as the cross‐sectional area of each fibre type as a percentage of the total fibre area. Finally, the non‐contractile tissue (NCT) percentage area was calculated by subtracting the total fibre area in the region of interest from the area of the region of interest, divided by the total area of the region of interest.

We used the method of capillary domains^22^ to analyze the capillarization in the muscle. Capillary co‐ordinates were taken from photographs of histological sections stained for capillaries with a digitizing tablet (Summagraphics model MMII 1201). The co‐ordinates were imported into AnaTis to calculate the capillary domains, defined as the area of a muscle cross‐section surrounding an individual capillary delineated by equidistant boundaries from neighbouring capillaries (*Figure* 2C). A capillary domain is a good estimate of the capillary oxygen supply area.^23^ This method not only provides overall indices of muscle capillarization, such as capillary density (number of capillaries per mm^2^) and the capillary‐to‐fibre ratio but also allows to determine the capillary supply to individual fibres even when they lack direct capillary contact (*Figure* 2D). The local capillary‐to‐fibre ratio (LCFR), defined as the sum of the fractions of the capillary domains overlapping an individual fibre, gives a continuous, rather than a discrete value of the capillary supply to a fibre and also takes into account that a capillary supplies more than one fibre. The capillary fibre density (CFD) is calculated as LCFR divided by the FCSA of the given fibre. Finally, the method of capillary domains gives a measure of the heterogeneity in capillary spacing [logarithmic standard deviation of the domain areas (Log_D_SD)].^3^

The staining intensity for SDH was determined as the optical density (SDH_OD (A_660_)) of the final reaction product using an interference filter of 660 nm at a magnification of ×10 (ImageJ, National Institute of Health, Bethesda, MD, USA) and was given as the absorbance at 660 nm (A_660_). In determining the OD of a fibre, the outline of the fibre was traced and the background OD subtracted. Fibres with freezing artefacts were excluded from the analysis. To minimize bias due to differences in lighting, for each section, a separate third‐order polynomial regression calibration curve was constructed with grey filters with a known OD. Van der Laarse *et al*.^24^ have shown that the maximal mass‐specific maximal oxygen uptake (VO_2_max_mass specific_) is proportional to the mitochondrial volume density and that the integrated SDH activity (SDH_INT = SDH_OD x FCSA) is linearly related with the maximum rate of oxygen uptake (VO_2_max_fibre_) or oxidative capacity of the muscle fibre.

VO_2_max_mass specific_ was expressed in L·kg^−1^·min^−1^ and calculated as follows:

$$VO_{2}\max\left(\text{mass specific}\right)=0.672\times \mathrm{SDH}\_\mathrm{OD}\left(A_{660}\right)$$

The maximal oxygen uptake of an individual muscle fibre (VO_2_max_fibre_ in pL·mm^−1^·min^−1^) was estimated from the VO_2_max_mass specific_ as follows:

$$VO_{2}\max\left(\text{fibre}\right)=0.672\times \mathrm{SDH}\_\mathrm{INT}=VO_{2}\max\left(\text{mass specific}\right)\times \text{FCSA}$$

The maximal oxygen consumption supported by a capillary (MO_2_max) was calculated as described previously^28^:

$$MO_{2}\max=\sum_{i=1}^{n}\;\left(VO_{2}\max\left(\text{mass specific}\right)*\text{Aovl}\right)$$

where Aovl is the area of each fibre within the capillary domain.

### Statistics

All data were analyzed with SPSS (Statistics version 21, IBM, Chicago, IL, USA), and *P* < 0.05 was taken to indicate a significant effect. Age and sex differences in anthropometric characteristics, FCSA and its respective standard deviation (SD FCSA), fibre type composition, NCT percentage, capillary supply indices that do not take into account fibre type, and MO_2_max were tested using a two‐way analysis of variance with age and sex as factors. In order to know whether type I and II fibres respond differently with age and sex, repeated measures analysis were performed for FCSA, SD FCSA, LCFR, CFD, VO_2_max_mass specific_, and VO_2_max_fibre_. If age × fibre type or sex × fibre type interactions were found, a two‐way analysis of variance with age and sex as factors was repeated for each fibre type separately. In testing for factors like FCSA, fibre type, and VO_2_max_mass specific_ that predict the capillary supply of the fibre (LCFR and CFD), a stepwise regression analysis was performed. Unless otherwise stated, results are presented as mean ± SD.

## Results

### Participant characteristics

Participant characteristics are given in *Table* 1. Women were shorter (*P* < 0.0005) and had a lower body mass than men (*P* = 0.001). Older participants were shorter than the young participants (*P* = 0.012), and with no significant age‐related differences in body mass, the body mass index was higher in the older participants than the younger participants (*P* = 0.004), irrespective of sex. The level of physical activity was not significantly different between young and old participants (although there was a tendency for higher activity in young participants; *P* = 0.054). Only 4 of the 26 older people had a physical activity score of 6 (indicating sedentary living), showing that the majority was recreationally active.

### Fibre size and shape

On average, 128 (32–311) fibres per participant were analyzed for fibre size. No hybrid fibres were detected in the muscle biopsies of 21 participants. In the others, hybrid fibres were excluded from further analysis as they represented only 2% of the fibre population.

**Figure 3:**
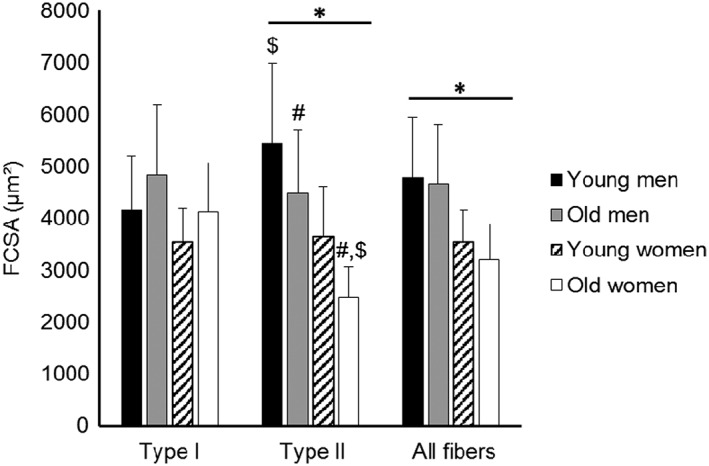
Fibre cross‐sectional area (FCSA) in the vastus lateralis muscle of young (*N* = 14) and old men (*N* = 22) and young (*N* = 5) and old women (*N* = 6) according to fibre type (type I and II and ‘all fibres’). Note that in ‘all fibres’, type I and II and hybrid fibres are included in the analysis. Values are mean ± SD; asterisk (*) indicates significant difference between men and women at *P* = 0.001; number sign (#) indicates significant difference from sex‐matched young people at *P* = 0.019; dollar sign ($) indicates type effect at *P* < 0.01.

The FCSA of all fibres combined was larger in muscles from men than in women (*P* = 0.001; *Figure* 3). A sex × fibre type interaction (*P* = 0.001) showed that while women had smaller type II fibres than men (*P* < 0.0005), the FCSA of type I fibres did not differ significantly between sexes. There was a significant age × fibre type interaction (*P* < 0.0005), indicating that the effects of age on FCSA differed between type I and II fibres. It can be seen that while the FCSA of type I fibres did not differ significantly between young and old participants, the FCSA of type II fibres was significantly smaller in older participants than younger participants, irrespective of sex (*P* = 0.019; *Figure* 3). There were no significant differences between fibre types, sex, or age in the fibre size variation, reflected by SD FCSA (*Table* 2).

**Table 2:** Fibre size variation and fibre type distribution in the vastus lateralis muscle

	Young		Old		Effects (*P*‐values)			Interactions (*P*‐values)		
	Men, *N* = 14	Women, *N* = 5	Men, *N* = 22	Women, *N* = 6						
					Age	Sex	Type	AS	AT	ST
SD FCSA All	1405 ± 461	1024 ± 173	1555 ± 455	1487 ± 540	0.056	0.158		0.322		
SD FCSA I and II	1201 ± 389	901 ± 267	1444 ± 446	1181 ± 422	0.068	0.053	0.132	0.817	0.172	0.926
SD FCSA I	1263 ± 322	863 ± 356	1501 ± 496	1320 ± 589	0.032	0.071		0.487		
SD FCSA II	1173 ± 414	936 ± 226	1404 ± 491	1081 ± 364	0.221	0.072		0.776		
FNP I (%)	44.5 ± 15.0	34.3 ± 9.5	44.6 ± 10.2	40.2 ± 8.4	0.471	0.077		0.481		
FNP II (%)	52.9 ± 15.2	65.4 ± 10.1	54.1 ± 11.0	58.6 ± 8.9	0.511	0.051		0.350		
FAP I (%)	38.1 ± 14.3	34.1 ± 7.3	45.7 ± 12.4	51.6 ± 13.7	0.007	0.826		0.269		
FAP II (%)	57.6 ± 14.9	65.4 ± 8.1	51.5 ± 13.3	45.7 ± 13.5	0.009	0.832		0.153		
Roundness I and II	1.28 ± 0.09	1.31 ± 0.07	1.36 ± 0.06	1.39 ± 0.06	0.002	0.308	<0.0005	0.908	0.002	0.019
Roundness I	1.31 ± 0.08	1.28 ± 0.06	1.34 ± 0.05	1.30 ± 0.03	0.132	0.115		0.845		
Roundness II	1.32 ± 0.04	1.33 ± 0.08	1.42 ± 0.08	1.47 ± 0.07	<0.0005	0.178		0.339		
NCT (%)	9.5 ± 2.8	10.5 ± 1.7	10.4 ± 2.8	10.7 ± 1.1	0.545	0.465		0.699		

I, type I fibres; II, type II fibres; AS, age × sex interaction; AT, age × fibre type interaction; FAP, fibre area percentage; FCSA, fibre cross‐sectional area; FNP, fibre number percentage; NCT, non‐contractile tissue; SD, standard deviation; ST, sex × fibre type interaction.

If the sum of fibre type proportions is less than 100%, this is due to hybrid fibres. Values are presented as mean ± standard deviation.

The shape factor of muscle fibres is given in *Table* 2. Overall, a higher deviation from circularity was observed in type II compared with type I fibres (*P* < 0.0005). There was a significant age × fibre type interaction (*P* = 0.002) for roundness, and it appeared that only type II fibres were less circular in the older muscle than young muscle (*P* < 0.0005). Finally, there was a positive correlation between Log_D_SD and FCSA SD (*R* = 0.302; *P* = 0.039) in our human muscle samples.

### Fibre type composition

The fibre type composition is given in *Table* 2. While no significant age effects were observed in the fibre number percentage of type I and II fibres, there was a tendency for a higher proportion of type II fibres in women than men (*P* = 0.051) and consequently a lower proportion of type I fibres in women (*P* = 0.077), irrespective of age. There was, however, no sex difference in the FAP occupied by the different fibre types. Irrespective of sex, the type II FAP was lower in the old people than the young people (*P* = 0.009) and conversely so for the type I FAP (*P* = 0.007), principally because of the smaller type II FCSA in muscles from older participants compared with young participants (*P* = 0.019; *Figure* 3). The percentage NCT did not differ significantly between the muscles from men and women, and those from young and old participants.

### Capillarization

On average, 224 (59–488) capillaries per participant were analyzed. Indices of muscle capillarization are given in *Table* 3. The capillary density, capillary‐to‐fibre ratio, and size of the capillary domain did not differ significantly between men and women, or young and older participants. In addition, the heterogeneity of capillary spacing, reflected by the Log_D_SD, was similar in all groups.

**Table 3:** Indices of capillary supply in the human vastus lateralis muscle

	Young		Old		Effects (*P*‐values)		Interactions (*P*‐values)
	Men, *N* = 13	Women, *N* = 5	Men, *N* = 22	Women, *N* = 6	Age	Sex	AS
CD (cap·mm^−2^)	331 ± 94	340 ± 120	286 ± 78	312 ± 139	0.284	0.606	0.795
C/F ratio	1.74 ± 0.57	1.44 ± 0.69	1.46 ± 0.41	1.08 ± 0.38	0.073	0.054	0.806
DOM (μm^2^)	3233 ± 1252	3149 ± 855	3667 ± 969	3434 ± 963	0.341	0.674	0.843
Log_D_SD	0.171 ± 0.022	0.166 ± 0.016	0.172 ± 0.018	0.158 ± 0.019	0.533	0.145	0.446

AS, age × sex interaction; CD, numerical capillary density; C/F ratio, ratio between the number of capillaries and number of fibres; DOM, capillary domain area; Log_D_SD, logarithmic standard deviation of the domain areas (representing heterogeneity of capillary spacing).

Data are presented as mean ± standard deviation.

**Figure 4:**
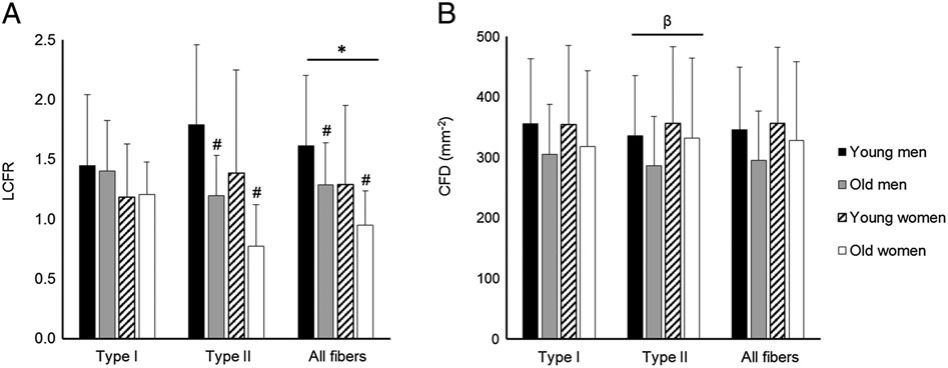
(A) Local capillary‐to‐fibre ratio (LCFR) and (B) capillary fibre density (CFD) in the vastus lateralis muscle of young (*N* = 13) and old men (*N* = 22), and young (*N* = 5) and old women (*N* = 6) according to the fibre type (types I and II and ‘all fibres’). Note that in ‘all fibres’, type I and II and hybrid fibres are included in the analysis. Values are mean ± SD; asterisk (*) indicates significant difference between men and women at *P* < 0.05; number sign (#) indicates significant difference from sex‐matched young people at *P* < 0.05; beta (β) indicates significant type × sex interaction reflected by a larger CFD of type II fibres in women than men.

The LCFR of all fibre types combined was lower in women than in men (*P* < 0.05; *Figure* 4A). A sex × fibre type interaction (*P* = 0.003) for CFD was reflected by the higher CFD of type II than type I in women, whereas it was the opposite in men.

There were significant age × fibre type interactions for LCFR (*P* < 0.0005). When the fibre types were analyzed separately, the LCFR of type II, but not type I fibres, was lower in older muscles than younger muscles (*P* < 0.01). These observations were explicable by the smaller size of the type II fibres in the older muscles, as the CFD did not differ significantly between young and old people, irrespective of fibre type and sex (*Figure* 4B).

### Succinate dehydrogenase activity

**Figure 5:**
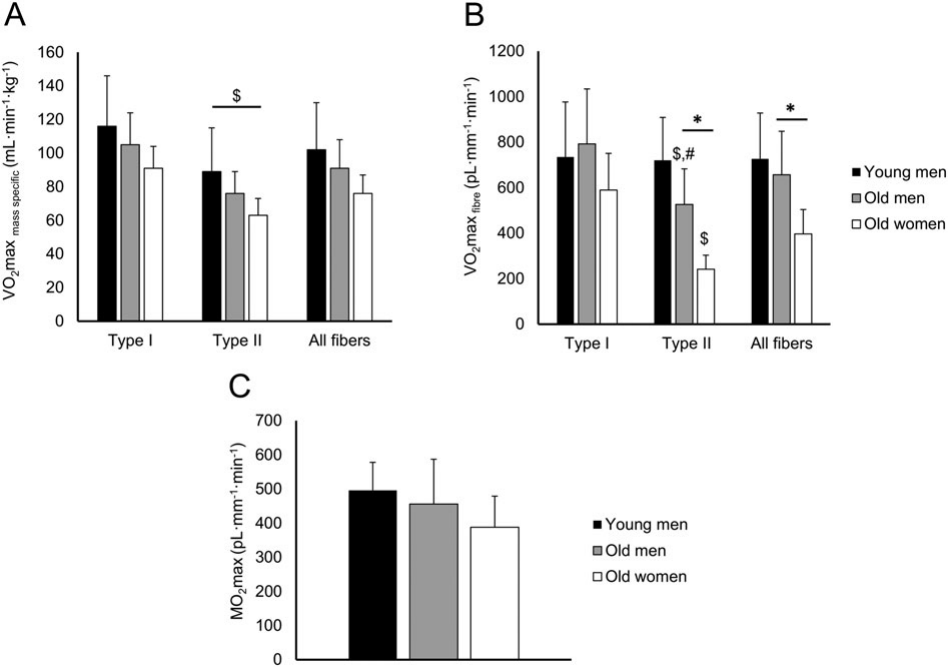
Fibre oxidative capacity (A) per unit muscle volume (VO_2_max _mass specific_) and (B) per mm fibre length (VO_2_max _fibre_) in the vastus lateralis muscle of young men (*N* = 5), old men (*N* = 14), and old women (*N* = 5) according to the fibre type (types I and II and ‘all fibres’). (C) Local maximal oxygen demand supported by a capillary (MO_2_max) in the vastus lateralis muscle of the same population. Note that in ‘all fibres’, type I and II and hybrid fibres are included in the analysis. Values are mean ± SD; asterisk (*) indicates significant difference between men and women at *P* < 0.05; number sign (#) indicates significant difference from sex‐matched young people at *P* < 0.05; dollar sign ($) indicates type effect at *P* < 0.01.

An average of 104 ± 48 fibres was analyzed for SDH activity per participant. There was, unfortunately, no muscle left to collect these data in young women. VO_2_max_fibre_ and VO_2_max_mass specific_ values in young men, old men, and old women for each fibre type are presented in *Table* 4 and *Figure* 5, respectively. VO_2_max_mass specific_ and VO_2_max _fibre_ were all higher in type I than type II fibres (*P* < 0.05; *Table* 4; *Figure* 5). There were no significant age or sex differences in VO_2_max_mass specific_ for each fibre type. There was, however, a significant fibre type × age interaction for VO_2_max_fibre_ (*P* = 0.010). This was reflected by a lower VO_2_max_fibre_ in old muscles than young muscles (*P* = 0.022) for type II, but not type I, fibres. Also, the VO_2_max_fibre_ was lower in women than men (*P* = 0.012). Differences in type II FCSA between men and women and young and old people, respectively, mainly explained these observations.

**Table 4:** Indices of oxidative capacity in the human vastus lateralis muscle

	Young men, *N* = 5	Old men, *N* = 14	Old women, *N* = 5	Effects (*P*‐values)			Interactions (*P*‐values)	
				Age	Sex	Type	AT	ST
VO_2_max_fibre_ All	727 ± 201	657 ± 191	397 ± 107	0.463	0.012			
VO_2_max_fibre_ I and II	727 ± 202	658 ± 191	398 ± 107	0.464	0.012	<0.0005	0.010	0.367
VO_2_max_fibre_ I	735 ± 242	793 ± 242	590 ± 161	0.635	0.103			
VO_2_max_fibre_ II	720 ± 188	526 ± 157	242 ± 62	0.022	0.002			
MO_2_max	496 ± 82	456 ± 131	388 ± 91	0.517	0.269			

I, type I fibres; II, type II fibres; MO_2_max, maximal oxygen consumption supported by a capillary (in pL·mm^−1^·min^−1^); VO_2_max_fibre_: maximal oxygen consumption of a fibre (in pL·mm^−1^·min^−1^). Data are presented as mean ± standard deviation.

**Figure 6:**
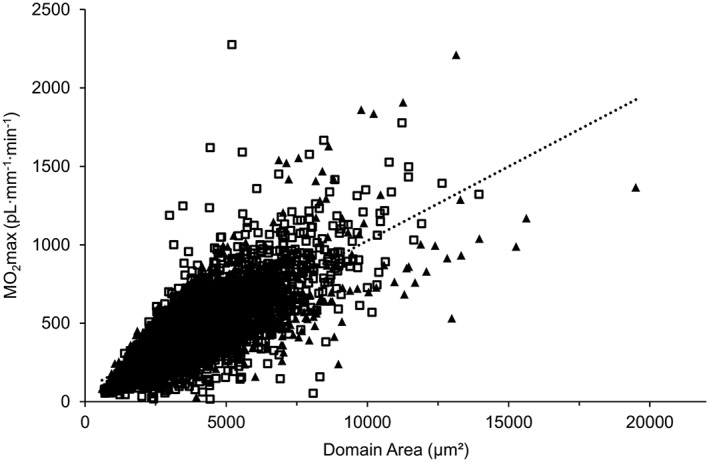
The relationship between the local muscle maximal oxygen demand supported by a capillary (MO_2_max) and their respective domain area in the vastus lateralis muscle. A positive correlation was observed between MO_2_max and domain area (*R* = 0.604, *N* = 4095 capillaries, *P* = 0.001). *R* = 0.854 ± 0.031 for regression lines from each young person (*N* = 6 individuals; black triangles) and *R* = 0.828 ± 0.018 for regression lines from each old man (*N* = 19 individuals; white square); mean ± SEM.

The estimated MO_2_max did not differ significantly between sex or age groups (*Table* 4), but there was a positive relationship between the capillary domain area and MO_2_max (*Figure* 6).

### Relationships between capillarization, succinate dehydrogenase activity, fibre cross‐sectional area, and fibre type

**Figure 7:**
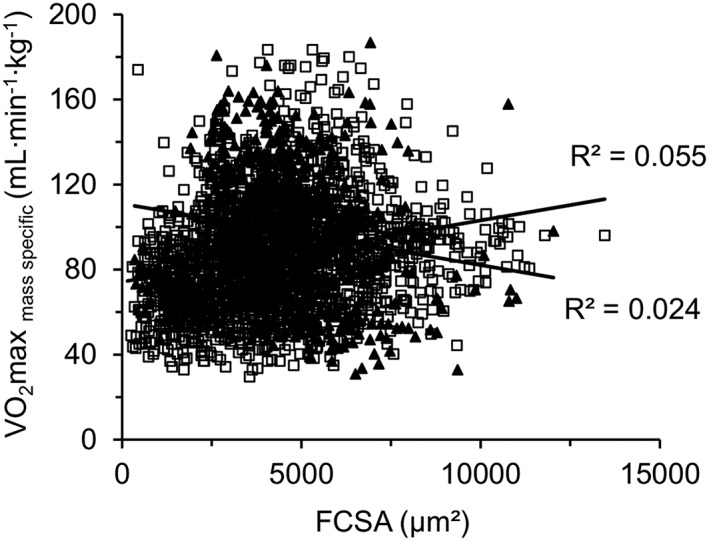
Fibre oxidative capacity per unit muscle volume (VO_2_max _mass specific_) in relation to fibre cross‐sectional area (FCSA) in young (*N* = 6 individuals; black triangles; *R* ^2^ = 0.024) and old men (*N* = 19 individuals; white squares; *R* ^2^ = 0.055) in the vastus lateralis muscle.

**Figure 8:**
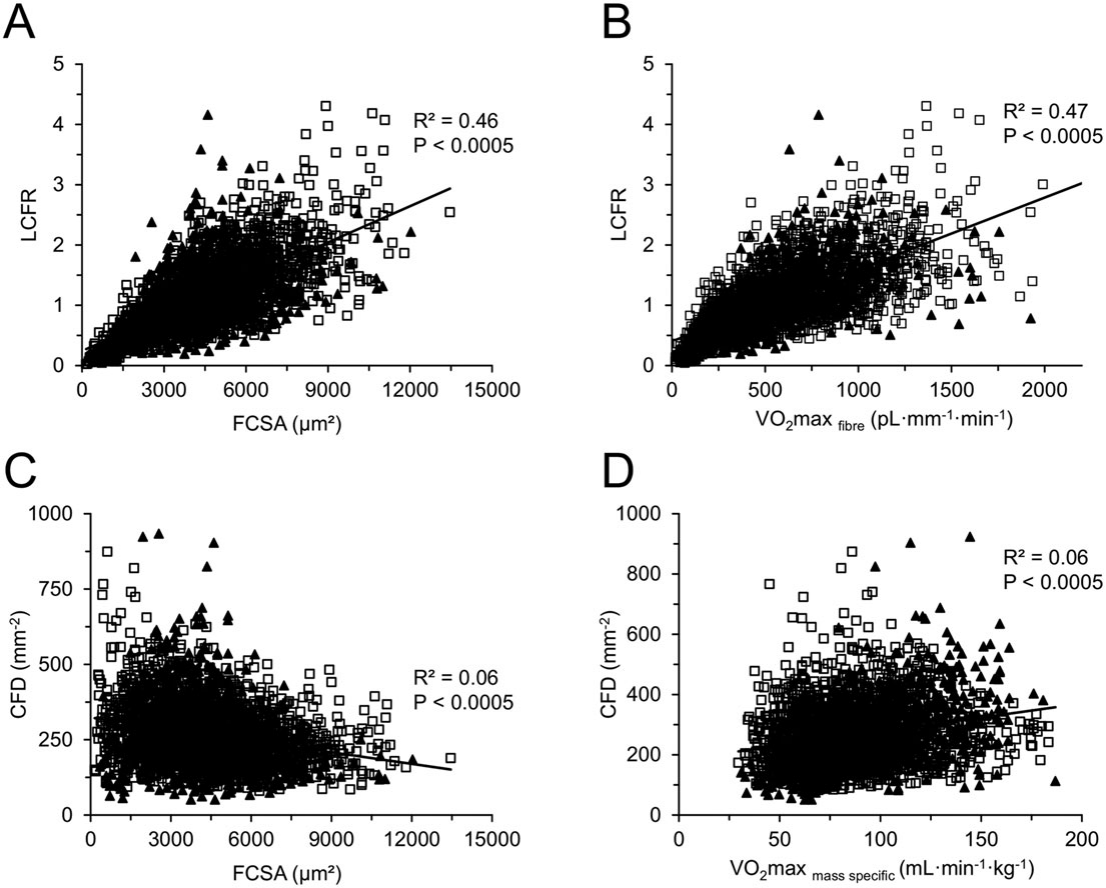
Relationships between the local capillary‐to‐fibre ratio (LCFR) with (A) the fibre cross‐sectional area (FCSA) and (B) the fibre oxidative capacity (VO_2_max _fibre_) in young (*N* = 6 individuals; black triangles) and old men (*N* = 19 individuals; white squares) in the vastus lateralis muscle. Relationships between the capillary fibre density (CFD) with (C) FCSA and (D) fibre oxidative capacity per muscle volume unit (VO_2_max _mass specific_) in young (*N* = 6 individuals; black triangles) and old men (*N* = 19 individuals; white squares) in the vastus lateralis muscle. Note that in (A) the FCSA and LCFR were significantly and positively correlated.

The VO_2_max_mass specific_ was not significantly related to FCSA (*Figure* 7). In both young and old muscles, the LCFR correlated positively with FCSA (*Figure* 8A). In assessing the contribution of different factors to the LCFR (capillary supply of a fibre), a stepwise linear regression was performed in which factors included sex, age, FCSA, VO_2_max_mass specific_, and fibre type. The primary determinant of LCFR was FCSA, which explained 46% of the variance in LCFR (*R* = 0.644; *P* < 0.0005). VO_2_max_mass specific_ and fibre type explained an additional 5.3% (*R* = 0.253; *P* < 0.0005) and 0.1% (*R* = 0.038; *P* < 0.018) of the variance in LCFR, respectively. There were no significant contributions of age or sex, suggesting that the qualitative and quantitative relationships between size and oxidative capacity of fibre with capillary supply are similar in men and women and do not change during healthy ageing.

## Discussion

The main observation of this study was that the age‐related preferential atrophy of type II fibres was accompanied by a decline in the number of capillaries supplying these fibres, such that the capillary density for type II fibres was similar in young and old, male and female muscles. There was no significant difference in the mass‐specific oxidative capacity of muscle fibres between young and old people. The similar quantitative and qualitative distribution of capillaries within muscles from healthy recreationally active older people and young adults indicates that the age‐related capillary rarefaction is not random, but maintains the coupling between skeletal muscle fibre size and capillarization during healthy ageing.

### Fibre size, fibre type composition, and shape factor

The capillary supply to a fibre was primarily determined by fibre size, and only to a small extent by the mass‐specific oxidative capacity in both young and aged muscles. In line with previous observations,^29^, ^30^, ^31^ men had larger—particularly type II—muscle fibres than women in the vastus lateralis muscle.^30^ While some studies reported no sex difference in muscle fibre type distribution,^31^, ^32^ we observed a tendency toward a higher proportion of type II fibres in women than men, as seen previously,^29^ but not when expressed as area percentage that takes the size of the fibres into account.

The approximately 35% lower muscle volume that we previously reported in this participant group^33^ was only partially explained by fibre atrophy. In agreement with many other studies,^34^, ^35^, ^36^ we found that the type II fibres were 18% smaller in muscles from the older participants than from the younger participants, while no such atrophy was observed for type I fibres. However, the similar average size of all fibres pooled in the old and the young participants and the greater variation in type I fibre size in the older participants suggest that the atrophy of type II fibres was accompanied by a concomitant (compensatory) hypertrophy, although not significant, of some type I fibres. We estimated up to 28% loss of muscle fibres *per se*, involving both types I and II fibres because the fibre type proportion did not differ between young and old people. It may be argued that this is an underestimation of the age‐related fibre loss, as it assumes that the whole muscle is built up of muscle fibres only, and it has been reported that the fat and connective tissue content in the muscle may increase with age.^37^ However, we did not find a significant difference in the percentage of NCT in the muscle biopsies. The increased variation in the size of type I fibres and incidence of angular type II fibres observed in our samples and others studying ageing,^27^ disuse,^2^ denervation,^38^ and reinnervation^39^ is likely a feature of the ongoing denervation–reinnervation process of motor unit remodelling.^40^ A 12 year follow‐up of older individuals also showed that the decrease in muscle volume was not associated with fibre atrophy,^41^ adding further evidence that fibre loss is the primary cause of the age‐related decrease in vastus lateralis muscle volume.^35^

### Mass‐specific oxidative capacity

The mass‐specific oxidative capacity, measured as the optical density of SDH‐stained muscle sections, is closely related to the fatigue resistance of the motor unit.^42^, ^43^ Values in type I fibres were around 45% greater than those in type II, but there were no age or sex differences in the oxidative capacity of fibres (reflecting the volume density of mitochondria) signifying a remarkable preservation in aged muscle in spite of the substantial muscle fibre morphological remodelling. Previous reports of age‐related reduction in the oxidative capacity from rat muscles^44^ and the human gastrocnemius^45^ may be due to an age‐related decrease in physical activity levels, whereas our physically and socially active participants likely benefited from activity‐related maintenance of oxidative capacity.^46^, ^47^

### Capillarization

There were no sex‐related differences in capillary density or capillary‐to‐fibre ratio. A lower number of capillaries supplying type II fibres in women compared with men was proportional to the smaller size of the type II fibres in women, because the CFD did not differ significantly between men and women.

While overall muscle capillarization, in terms of capillary density and heterogeneity of capillary spacing, was similar in young and old people, the number of capillaries per type II fibre (LCFR) was 38% lower in the old muscles than in the young muscles, which is indicative of capillary rarefaction. The loss of capillaries occurred primarily around type II fibres as has also been seen by others,^48^ while the capillary supply per unit type II fibre area was not affected by age. This indicates that the loss of capillaries was proportional to the atrophy of type II fibres. As discussed previously, there might have occurred a 28% loss of muscle fibres, probably because of an incomplete reinnervation of fibres denervated consequent to motor neuron loss in old age.^49^ Such a loss of fibres without capillary rarefaction would result in an increased capillary‐to‐fibre ratio or LCFR, something we did not observe. Capillary rarefaction during ageing thus appears to be proportional to the combined decrease in fibre size and fibre loss.

The close relationship between capillary supply and fibre size, but less so for fibre oxidative capacity, in young and aged muscles^5^, ^6^, ^7^, ^28^ as well as the reported similar time course of fibre hypertrophy and angiogenesis during the development of compensatory hypertrophy^8^ indicate that the size and capillary supply of a fibre share similar control mechanisms. In fact, both endothelial cells and muscle cells are mechanosensitive, and each secrete factors that stimulate muscle growth and angiogenesis.^50^

One factor that has previously not been considered in studies of ageing human muscle is the heterogeneity of capillary spacing, reflected by the logarithmic standard deviation of the capillary supply areas (Log_D_SD).^22^ An increase in the heterogeneity of capillary spacing has an adverse impact on tissue oxygenation.^15^, ^16^, ^17^, ^19^, ^51^, ^52^ Here, we found in human muscle, similar to the observation in rat muscle,^20^ that the heterogeneity of capillary spacing is related to the variability in fibre size, as reflected by the positive correlation between Log_D_SD and FCSA SD in the human study. Even though the FCSA SD of type I fibres was higher in the older muscles, it is striking that the heterogeneity of capillary spacing was maintained, considering also there must have been significant capillary rarefaction as reflected by the maintained capillary density in the face of an up to 28% loss of muscle fibres. The similar Log_D_SD in young and old people indicates that the capillary rarefaction during ageing does not occur at random, but rather maintains the distribution of capillaries to preserve the potential for adequate intramuscular oxygenation. The capillary rarefaction was evident in the absence of fibre atrophy in a 12 year follow‐up study,^41^ which suggests that capillary rarefaction is a prelude to age‐related fibre atrophy.

Even though the anatomical capillary supply may be similar in young and old people, this does not necessarily mean that the maximal oxygen delivery to the muscle and muscle fibres is also maintained in old age. It has been shown for instance that the vasodilatory response during exercise, and hence blood flow to the muscle, decreases with age.^53^, ^54^ Because shear stress plays an important role in the maintenance of the capillary bed and angiogenesis,^1^ it may well be that this impaired vasodilatory response underlies the progressive, gradual loss of capillaries during ageing. If so regular physical activity, which will increase muscle blood flow and hence endothelial shear stress, may prevent some of the age‐related capillary rarefaction. Indeed, in master athletes, the capillary‐to‐fibre ratio was larger than in activity‐matched young controls,^47^ while in sedentary older people the capillary‐to‐fibre ratio was reduced.^45^

### Relationships between the capillary supply to a fibre and its size, type, and oxidative capacity

An inverse relationship between fibre size and oxidative metabolism has been suggested.^55^ However, our results challenge this finding by showing no inverse relationship between FCSA and VO_2_max_mass specific_ (*Figure* 7) and are in line with our previous work showing that considerable hypertrophy can develop without, as predicted by this concept, concomitant decrease in the mass‐specific oxidative capacity of muscle fibres.^56^ A rather surprising finding was that in muscles from young people,^28^ and also in those from older people, the maximal oxygen consumption supported by a capillary varies more than 100‐fold between capillaries (*Figure* 6). This indicates that the local muscle capillarization is not necessarily matched to local oxidative capacity.^28^ Mitochondria may not work maximally during contractile activity, and a heterogeneous capillary perfusion^57^, ^58^ affecting tissue oxygenation^59^ may help match oxygen demand and delivery. Such a functional connectivity between active muscle fibres, their surrounding capillaries and the arterioles is well known.^60^, ^61^ Nevertheless, the 100‐fold variation in maximal oxygen demand supported by a capillary deviates from the concept of symmorphosis, which states that structures and demand are matched.^62^ An explanation proposed by Wüst *et al*.^7^ for such a phenomenon is that tight packing of subsarcolemmal mitochondria in close proximity to capillaries leads to non‐homogeneous mitochondrial distribution within muscle fibres, particularly those with high mitochondrial density, which was also evident in our samples (data not shown) and previous studies.^63^, ^64^

### Study limitations

From the cross‐sectional design of our study, it is not possible to determine whether changes in muscle capillarization with ageing precede or follow changes in muscle fibre size and number. A 12 year longitudinal study showing capillary rarefaction without fibre atrophy^41^ suggests that rarefaction may precede atrophy during ageing. Muscle biopsies by definition provide only a small sample of the whole muscle and can introduce a sampling bias, at least partly related to differences in fibre type composition over the depth of the muscle.^65^ To minimize this latter bias, we have taken all biopsies from a similar location in the mid‐muscle belly of the vastus lateralis muscle, determined by distances from landmarks, and depth. Finally, it is possible that the SDH activity does not fully reflect changes in oxidative capacity in aged muscles because ageing may be associated with greater reductions in the activity of electron transport chain complexes containing mitochondrial DNA encoded subunits (e.g. complexes I, III, IV, and V)^66^ than that of mitochondrial enzymes that are entirely nuclear DNA encoded (e.g. complex II).^67^ Such a situation would be reflected in muscle biopsies by fibres with an increased activity of SDH, as a compensation for, for example, the reduction in cytochrome oxidase (complex IV) activity.^68^ It is unlikely that such a situation would bias our data significantly as the SDH activity in muscle fibres was similar in young and old muscles (Figure 5A).

## Conclusions

The main observation of the present study is that in recreationally active older adults with similar physical activity levels as younger adults, there is no significant decrease in muscle fibre oxidative capacity, but significant type II fibre atrophy and capillary rarefaction. Despite the fibre atrophy, fibre loss, and capillary rarefaction, the relationship between capillary supply and fibre size was maintained in the old people. The similar capillary distribution indicates that the capillary rarefaction during ageing does not occur at random, but maintains the distribution of capillaries to preserve the potential for intramuscular oxygenation.

## Funding

This work was supported in part by the European Union within the FP7 Project ‘Myoage’ (contract no 23576), the Association Française contre les Myopathies, and the Research Councils UK the Lifelong Health and Wellbeing cross‐Council initiative (MR/K025252/1).

## Conflict of interest

The authors declare that they have no conflicts of interest.
